# Immune cell population and cytokine profiling suggest age dependent differences in the response to SARS-CoV-2 infection

**DOI:** 10.3389/fragi.2023.1108149

**Published:** 2023-02-13

**Authors:** Larraitz Aragon, Andrea Iribarren-López, Ainhoa Alberro, Leire Iparraguirre, Miguel Von Wichmann, Jose María Marimon, Nagore Saiz-Calderon, Julia Agudo, M. Isabel Gálvez, M. Carmen Cipitria, Alvaro Prada, David Otaegui

**Affiliations:** ^1^ UGC Laboratories Gipuzkoa, Immunology Section, Osakidetza Basque Health Service, San Sebastián, Spain; ^2^ Multiple Sclerosis Group, Neurosciences Area, Biodonostia Health Research Institute, San Sebastián, Spain; ^3^ Infectious diseases Department, Donostialdea Integrated Health Organization, Osakidetza Basque Health Service, San Sebastián, Spain; ^4^ Microbiology Department, Donostialdea Integrated Health Organization, Osakidetza Basque Health Service, San Sebastián, Spain

**Keywords:** COVID-19, SARS-CoV-2, immunosenescence and exhaustion, immunosenescence and inflammaging, Severe COVID-19

## Abstract

Aging population is at higher risk of developing severe COVID-19, including hospitalization and death. In this work, to further understand the relationship between host age-related factors, immunosenescence/exhaustion of the immune system and the response to the virus, we characterized immune cell and cytokine responses in 58 COVID-19 patients admitted to the hospital and 40 healthy controls of different age ranges. Lymphocyte populations and inflammatory profiles were studied in blood samples, using different panels of multicolor flow cytometry. As expected, our analysis reveals differences at both the cellular and cytokine level in COVID-19 patients. Interestingly, when the age range analysis was carried out, the immunological response to the infection was found to differ with age, being especially affected in the group of 30–39 years. In this age range, an increased exhausted T cell response and a decrease of naïve T helper lymphocytes was found in patients, as well as a reduced concentration of the proinflammatory TNF, IL-1β and IL-8 cytokines. Besides, the correlation between age and the study variables was evaluated, and multiple cell types and interleukins were found to correlate with donor age. Notably, the correlations of T helper naïve and effector memory cells, T helper 1–17 cells, TNF, IL-10, IL-1β, IL-8, among others, showed differences between healthy controls and COVID-19 patients. Our findings, in the context of other previous studies, suggest that aging affects the behavior of the immune system in COVID-19 patients. They suggest that young individuals are able to mount an initial response to SARS-CoV-2, but some of them present an accelerated exhaustion of the cell response and an insufficient inflammatory response, resulting in a moderate to severe COVID-19. On the other hand, in older patients there is a smaller immune cell response to the virus, reflected in fewer differences in immune populations between COVID-19 patients and controls. Nevertheless, old patients show more evidence of an inflammatory phenotype, suggesting that the underlying inflammation associated with their age is exacerbated by the SARS-CoV-2 infection.

## 1 Introduction

Coronavirus disease 2019, better known as COVID-19, is an infectious disease produced by the Severe Acute Respiratory Syndrome Coronavirus 2 (SARS-CoV-2). SARS-CoV-2 is a novel strain in the coronavirus family, a group of enveloped, positive-sense, single stranded RNA viruses. The first outbreak was reported in December 2019 in a seafood market in Wuhan, China, after a likely zoonotic spillover event (Zhu et al., 2020). From then on, and due to the efficient human-to-human transmission, SARS-CoV-2 infection spread across the globe so rapidly that by March 2020, it resulted in an unprecedented public health crisis worldwide and the World Health Organization officially declared COVID-19 as a pandemic (Li et al., 2020).

SARS-CoV-2 infects people regardless of their age, sex and ethnicity, but COVID-19 disease presentation is markedly heterogeneous, with different symptoms and outcomes (Cascella et al., 2022). For most of the cases, clinical manifestations range from being asymptomatic to having mild upper respiratory illness that manifests as fever, cough, shortness of breath, anosmia, myalgia, headache, and/or fatigue. Nevertheless, a proportion of patients will suffer severe viral pneumonia and respiratory failure–requiring supplemental oxygen or mechanical ventilation–as well as metabolic acidosis, coagulopathy, septic shock and multiple-organ failure that can ultimately be fatal (Cascella et al., 2022).

Growing evidence indicates that while COVID-19 includes an initial stage of viral replication, it is followed by a second stage of immunopathology driven by a hyperinflammatory response to SARS-CoV-2 (Gustine and Jones, 2021). This hyperinflammatory syndrome has been associated with a dysregulated host immune response characterized by abnormal responses both at molecular and cellular level such as a “cytokine storm” with abnormally elevated levels of IL-1, IL-6, IL-8 and TNF-alpha among others, lymphopenia, dysregulated macrophage activation, impaired natural killer cell response, increased proportions of activated T cell producing cytokines and elevated neutrophil count (Del Valle et al., 2020; Giamarellos-Bourboulis et al., 2020; Hadjadj et al., 2020; Huang and Pranata, 2020; Laing et al., 2020; Lucas et al., 2020; Ronit et al., 2020; Caricchio et al., 2021; Masso-Silva et al., 2022). Moreover, several studies indicate that the hyperinflammatory syndrome contributes to disease severity and mortality (Del Valle et al., 2020; Hadjadj et al., 2020; Laing et al., 2020; Caricchio et al., 2021), suggesting that the homeostasis of the immune system plays a major role in COVID-19 outcome.

In addition, data indicate that older patients are at higher risk of developing severe COVID-19, including hospitalization and death. In fact, based on the reports published by the Spanish National Epidemiological Surveillance Network (RENAVE) the 84.3% of the SARS-CoV-2 positive patients that died by March 2022 in Spain were patients older than 70 years while they only represented the 9.2% of the infections (Red Nacional de Vigilancia Epidemiológica, 2022). This evidence suggests that there are host-related factors that make older individuals particularly vulnerable to more severe outcomes of the SARS-CoV-2 infection. Nevertheless, the reasons for this increased vulnerability in older individuals are still not fully understood.

From an immunological viewpoint, it is already known that with aging, the immune system suffers numerous changes affecting nearly every component of both innate and adaptive immune responses (Aiello et al., 2019). These changes have been gathered under the term immunosenescence (Pawelec, 2012; Nikolich-Žugich, 2018). Three of the most relevant processes of immunosenescence are the increase in the memory/naïve cell ratio (Kim et al., 2008; Pawelec, 2012), the shrinkage of the T cell repertoire caused by thymic involution (Lynch et al., 2009) and the chronic low-grade inflammation (Franceschi and Campisi, 2014; Alberro et al., 2021). These changes have been related to a decreased ability to respond to new antigens and an increased vulnerability to infectious diseases, as well as increased morbidity and mortality due to infections (Kauffman, 2004). Interestingly, immunosenescence does not fit perfectly with biological age, as it is affected by many other variables.

In the last 2 years, several studies have characterized the cellular immune response and cytokine production in response to SARS-CoV-2 infection and reported a sharp decrease in lymphocytes (Huang and Pranata, 2020) as well as the previously described hyperinflammatory state (Gustine and Jones, 2021). A number of these studies have evaluated senescence/exhaustion markers such as CD57, PD-1, Tim-3, NKG2A and CTLA-4 in order to understand how the SARS-CoV-2 infection affects the exhausted phenotype of the immune system. All of them found that patients had higher percentages of T helper and cytotoxic senescent/exhausted cells (Zheng et al., 2020a; Zheng et al., 2020b; De Biasi et al., 2020; Diao et al., 2020; Song et al., 2020; Rha et al., 2021). Moreover, some of the studies found that there is a progressive T cell exhaustion, particularly of cytotoxic T cell, with severity of COVID-19 (Diao et al., 2020; Song et al., 2020), and that after antiviral therapy (Zheng et al., 2020b) or after negative conversion of SARS-CoV-2 the levels of exhausted cells decrease (Rha et al., 2021).

Nevertheless, many aspects of immune response and the effect of age on COVID-19 disease remain unclear. Therefore, in this study, in order to further understand the relation between host age-related factors, immunosenescence/exhaustion of the immune system and the response to the virus, we have characterized immune-cell and cytokine responses in COVID-19 patients and healthy controls of different age ranges.

## 2 Materials and methods

### 2.1 Participants and sample extraction

This study has been conducted at the Donostia University Hospital (HUD) as a collaboration between the Immunology, Microbiology and Infectious diseases departments together with the Multiple Sclerosis group of Biodonostia Health Research Institute and the Basque Biobank (www.biobancovasco.org). The study was approved by the Donostia University Hospital’s ethics committee (PI2020076) and all donors provided written informed consent for blood sampling and analysis of their clinical data records.

For the characterization of immune cell populations, 58 patients diagnosed with SARS-CoV-2 infection and 40 healthy controls were enrolled. SARS-CoV-2 infection was confirmed by detection of viral RNA in specimens from the patients’ nasopharyngeal swabs by RT-qPCR at the HUD Microbiology department. All the COVID-19 donors included in the study were admitted to the hospital due to their symptoms (respiratory distress ≧30 breaths/min, or oxygen saturation ≤93% at rest, or arterial partial pressure of oxygen/fraction of inspired oxygen ≦300 mmHg) and, therefore, samples come from moderate and severe cases. COVID-19 patients and healthy donors with previous immune-related diseases were excluded from the study. None of the COVID-19 patients included in the study progressed to ICU admission. The volunteers enrolled as healthy controls did not present any symptoms or pathology. In addition, they did not suffer from any kind of immune-mediated disease that could interfere with the results.

5 mL of whole blood were obtained from all individuals by venipuncture in EDTA tubes (Vacuntainer, BD Biosciences). For the study of circulating cytokines, apart from the 58 COVID-19 and the 40 healthy controls, 29 additional COVID-19 patients were included. Serum samples were obtained from silicone coated serum tubes (Vacuntainer, BD Biosciences) after centrifugation at 1000 g for 10 min. Data of the first blood analysis after the COVID-19 diagnosis, when patients were admitted to the hospital, were retrieved from the clinical records and correctly anonymized. The main demographic data of all the individuals enrolled in the study are shown in Table 1.

**TABLE 1:** Main demographic characteristics of individuals enrolled in the study classified by the two different cohorts that have been studied.

Cohort	Group	Sex	Mean age (SD)
Immune cells populations	COVID-19 patients (n = 58)	Female (n = 26)	52.2 (±12.6)
		Male (n = 32)	53.1 (±15.2)
	Healthy controls (n = 40)	Female (n = 33)	49.1 (±11.2)
		Male (n = 7)	52 (±13.4)
Cytokines	COVID-19 patients (n = 87)	Female (n = 41)	52.9 (±12.2)
		Male (n = 46)	56.7 (±14.5)
	Healthy controls (n = 40)	Female (n = 33)	49.1 (±11.2)
		Male (n = 7)	52.0 (±13.4)

Patients’ and healthy controls’ ages ranged from 21 to 79 years and for the analysis by age ranges, they were classified in four groups including those aged from 30 to 39 years, 40–49 years, 50–59 years and 60–69 years. Individuals younger than 30 and older than 69 years were excluded from this analysis to guarantee equilibrated age ranges with a minimum number of 8 samples per group. The main demographic data of the individuals classified by age ranges are shown in Table 2.

**TABLE 2:** Main demographic characteristics of individuals enrolled in the study separated by age ranges and classified by the two different cohorts that have been studied.

	Immune cell populations			Cytokines		
Age range	Group	Sex	Mean age (SD)	Group	Sex	Mean age (SD)
30–39 years	COVID-19 patients (n = 9)	Female (n = 6)	34.5 (±2.3)	COVID-19 patients (n = 9)	Female (n = 6)	34.5 (±2.3)
		Male (n = 3)	36.6 (±1.1)		Male (n = 3)	36.6 (±1.1)
	Healthy controls (n = 9)	Female (n = 7)	33.9 (±3.3)	Healthy controls (n = 9)	Female (n = 7)	33.9 (±3.3)
		Male (n = 2)	35.0 (±2.8)		Male (n = 2)	35.0 (±2.8)
40–49 years	COVID-19 patients (n = 18)	Female (n = 3)	45.7 (±2.5)	COVID-19 patients (n = 22)	Female (n = 7)	45.1 (±2.8)
		Male (n = 15)	45.3 (±2.8)		Male (n = 15)	45.3 (±2.8)
	Healthy controls (n = 8)	Female (n = 7)	46.7 (±2.8)	Healthy controls (n = 8)	Female (n = 7)	46.7 (±2.8)
		Male (n = 1)	45.0		Male (n = 1)	45.0
50–59 years	COVID-19 patients (n = 13)	Female (n = 10)	54.3 (±3.1)	COVID-19 patients (n = 23)	Female (n = 16)	54.4 (±2.8)
		Male (n = 3)	55.3 (±2.3)		Male (n = 7)	55.3 (±2.5)
	Healthy controls (n = 11)	Female (n = 11)	53.9 (±3.4)	Healthy controls (n = 11)	Female (n = 11)	53.9 (±3.4)
		Male (n = 0)	-		Male (n = 0)	-
60–69 years	COVID-19 patients (n = 9)	Female (n = 6)	65.3 (±3.4)	COVID-19 patients (n = 16)	Female (n = 8)	65.3 (±2.9)
		Male (n = 3)	63.7 (±4.7)		Male (n = 8)	63.5 (±3.5)
	Healthy controls (n = 11)	Female (n = 7)	62.7 (±1.7)	Healthy controls (n = 11)	Female (n = 7)	62.7 (±1.7)
		Male (n = 4)	62.3 (±2.6)		Male (n = 4)	62.3 (±2.6)

### 2.2 Lymphocyte count and flow cytometry

Absolute leukocyte counts (CD45^+^) and T lymphocyte (CD3^+^), T helper (Th) lymphocyte (CD4^+^), T cytotoxic (Tc) lymphocyte (CD8^+^), B lymphocyte (CD19^+^), and NK cell (CD16/56+) subsets were determined in a total of 50 µL of the whole blood samples using BD Multitest™ 6-color TBNK Reagent kit with BD Trucount tubes (BD Biosciences, #337166) following the manufacturer’s instructions. A FACS Canto II flow cytometer (BD Biosciences) was used for acquisition. Results were manually checked using Infinicyt analysis software (Cytognos).

T lymphocyte subpopulations and senescence and exhaustion markers were studied using different panels of multicolor flow cytometry (Table 3). To do so, the remaining whole blood sample was partitioned in four tubes with 250 µL each and incubated with the antibodies shown in Supplementary table 1 for 20 min in the dark at room temperature. After incubation with the corresponding antibodies cells were lysed with 2 mL of FACS Lysing solution (BD Biosciences) for 10 min and washed with 2 mL of FACSFlow (BD Biosciences). Finally, cells were resuspended in 450 μL of FACS Flow and analysed in a FACS Canto II flow cytometer (BD Biosciences) using the FACS Diva acquisition software (BD Biosciences). The gating strategy for subpopulation analysis and the flow cytometry plots are shown in Supplementary Figure 1. Results were checked manually using Infinicyt analysis software (Cytognos).

**TABLE 3:** List of immune cell populations analysed by flow cytometry and their corresponding markers.

Immune cell population subsets	Markers^a^
Total leukocytes (%)	CD45+
T lymphocytes (%)	CD3+
T helper lymphocytes (%)	CD4+
Naive T helper lymphocytes (%)	CD45RA+CD197+
Central memory T helper lymphocytes (%)	CD45RA−CD197+
Effector memory T helper lymphocytes (%)	CD45RA−CD197−
Terminally differentiated T helper lymphocytes (%)	CD45RA+CD197−
T helper type 1 lymphocytes (Th1) (%)	CD196−CD183+
T helper type 2 lymphocytes (Th2) (%)	CD196−CD183−
T helper type 17 lymphocytes (Th17) (%)	CD196+CD183−
T helper type 1–17 lymphocytes (Th1–17) (%)	CD196+CD183+
Regulatory T lymphocytes (%)	CD25+CD127−
Exhausted T helper lymphocytes (%)	PD−1+
Senescent T helper lymphocytes (%)	CD28−CD57+
T cytotoxic lymphocytes (%)	CD8+
Naive T cytotoxic lymphocytes (%)	CD45RA+CD197+
Central memory T cytotoxic lymphocytes (%)	CD45RA−CD197+
Effector memory T cytotoxic lymphocytes (%)	CD45RA−CD197−
Terminally differentiated T cytotoxic lymphocytes (%)	CD45RA+CD197−
Exhausted T cytotoxic lymphocytes (%)	PD−1+
Senescent T cytotoxic lymphocytes (%)	CD28−CD57+
B lymphocytes (%)	CD19+
Natural Killer cells (%)	CD16+CD56+

a Note that they have been classified by subfamilies and the general marker for the fasmily (such as the CD3^+^ for all the T lymphocytes) has been omitted for more clarity.

### 2.3 Cytokine measurement

Serum levels of Interleukin-8 (IL-8), Interleukin-1β (IL-1β), Interleukin-6 (IL-6), Interleukin-10 (IL-10), Tumor Necrosis Factor (TNF), and Interleukin-12 (IL-12) were determined by flow cytometry using the BD CBA Human Inflammatory Cytokines Kit (BD Biosciences, #551811) following manufacturer’s instructions. A FACS Canto II flow cytometer (BD Biosciences) and the FACS Array software (BD Biosciences) were used for the analysis.

### 2.4 Statistical analysis

IBM SPSS version 23 (IBM Corporation, 2015) and R-Studio (RStudio Team, 2015) with R version 4.1.2 statistical software were used for the statistical analyses. Immune cell population variables were analyzed as percentage and cytokine variables as concentration in pg/ml. Shapiro-Wilk was used to test for normality. Taking into account the differences in the number of female and male individuals across groups, all the mean comparisons were corrected by sex as a covariate. ANCOVA and Non-parametric ANCOVA tests were used for mean comparisons between groups for normally and non-normally distributed variables correspondingly. Pearson and Spearman correlation tests were conducted to assess the association between age and normally and non-normally distributed immune variables respectively. For all the analyses significance was set at *p*-value<0.05. Corrections for multiple testing were not performed.

## 3 Results

### 3.1 Global immunological changes are found in COVID-19

Eighty-seven COVID-19 patients who were admitted to the Donostia University Hospital between September 2020 and June 2021 and 40 volunteers, whose samples served as healthy controls, were recruited for the study. After confirmation of the SARS-CoV-2 infection by RT-qPCR in nasopharyngeal samples, we assessed their cytokine serum levels and immune cell profiles by flow cytometry in freshly isolated blood samples.

Basic demographic information is shown in Tables 1, 2, where it can be observed that there is no significant difference in ages between groups (*p* = 0.08). However, the proportion of women is significantly higher (*p* < 0.0001) in the healthy control group (82.5%) when compared to the patients’ group (47.1%), and for that reason, all the analyses have been corrected by sex as a covariate.

**FIGURE 1:**
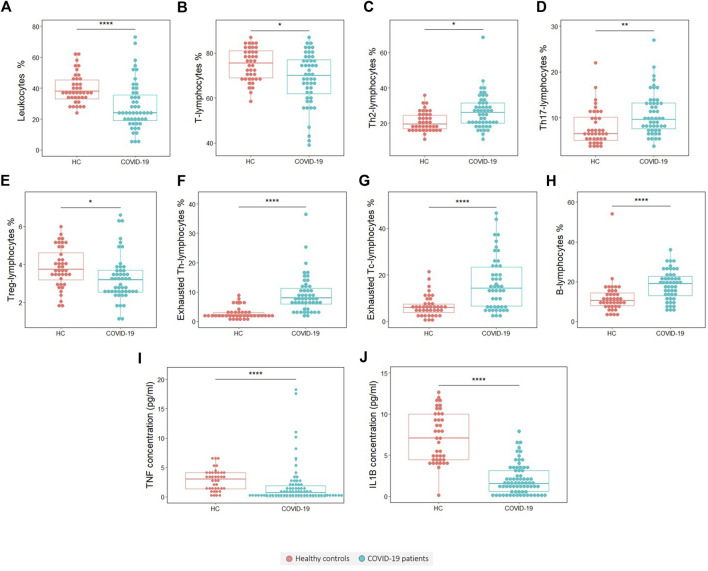
Variables found to be significantly different in COVID-19 patients with respect to healthy controls (HCs). **(A)** Leukocytes percentage, **(B)** T lymphocytes percentage, **(C)** Th2 lymphocytes percentage, **(D)** Th17 lymphocytes percentage, **(E)** T regulatory lymphocytes percentage, **(F)** Exhausted Th lymphocytes percentage, **(G)** Exhausted Tc-lymphocytes percentage, **(H)** B lymphocytes percentage, **(I)** TNF concentration and **(J)** IL-1β concentration. The statistical significance is depicted with asterisk code meaning: *p < 0.05; **p < 0.01; ***p < 0.001; ****p < 0.0001.

Flow cytometry results for the comparisons between the 58 COVID-19 patients and 40 healthy controls are shown in Table 4. Moreover, variables with significant differences are plotted in Figure 1. The patient group exhibited a significantly lower proportion (*p* < 0.0001) and absolute number (*p* < 0.0001) of leukocytes than the control group (Figure 1A and Supplementary Table 2). This difference goes together with a significant reduction in the proportion of T lymphocytes (*p* = 0.03) (Figure 1B), whereas the percentage of B lymphocytes is significantly increased in patients (*p* < 0.0001) (Figure 1H). The proportion of NK cells does not change with the disease, although we recorded a reduction in the absolute number of NK cells in patients (Supplementary Table 2). Similarly, the proportion of T helper and T cytotoxic lymphocytes is not affected in patients, while the absolute cell number of these populations is decreased in COVID-19 patients (*p* < 0,0001 in both cases) (Supplementary Table 2). Regarding the subsets of T helper lymphocytes, it can be observed that patients present a higher tendency to cell differentiation to Th2 (anti helminth response) (*p* = 0.034) (Figure 1C) and Th17 (anti fungi and extracellular bacteria response) (*p* = 0.003) (Figure 1D) subtypes and a slight but significant decrease in regulatory T lymphocytes (*p* = 0.033) (Figure 1E).

**TABLE 4:** Statistical analysis of immune cell populations and cytokine concentration results. Mean and standard deviation results are shown for each of the variables and groups.

	Healthy controls	COVID-19 patients	Fold change	*p*-value
**Total leukocytes (%)**	**39.65 (±9.4)**	**27.17 (±15.1)**	**−1.31**	**<0.0001**
**T lymphocytes (%)**	**74.83 (±7.4)**	**67.95 (±12.2)**	**−1.09**	**0.033**
T helper lymphocytes (%)	49.63 (±7.7)	44.09 (±11.6)	−1.09	0.147
Naive T helper lymphocytes (%)*	40.93 (±16.4)	39.36 (±14.8)	−1.08	0.362
Central memory T helper lymphocytes (%)	34.84 (±11.9)	36.77 (±11.7)	1.11	0.063
Effector memory T helper lymphocytes (%)	20.91 (±10.8)	20.80 (±9.5)	−1.03	0.945
Terminally differentiated T helper lymphocytes (%)	4.07 (±5.8)	3.15 (±5.7)	−1.23	0.326
T helper type 1 lymphocytes (Th1) (%)	18.95 (±11.8)	15.20 (±6.5)	−1.17	0.472
** ** **T helper type 2 lymphocytes (Th2) (%)**	**21.04 (±5.5)**	**26.41 (±9.6)**	**1.20**	**0.034**
**T helper type 17 lymphocytes (Th17) (%)**	**7.75 (±4.0)**	**10.70 (±5.0)**	**1.36**	**0.003**
T helper type 1–17 lymphocytes (Th1-17) (%)	12.19 (±7.0)	9.98 (±6.0)	−1.13	0.375
**Regulatory T lymphocytes (%)**	**3.85 (±1.1)**	**3.37 (±1.3)**	**−1.12**	**0.032**
**Exhausted T helper lymphocytes (%)**	**2.78 (±2.0)**	**9.72 (±6.6)**	**3.44**	**<0.0001**
Senescent T helper lymphocytes (%)	2.72 (±5.9)	1.86 (±2.5)	−1.37	0.659
T cytotoxic lymphocytes (%)	23.60 (±9.4)	21.69 (±9.3)	−1.17	0.165
Naive T cytotoxic lymphocytes (%)	27.11 (±15.8)	26.30 (±16.2)	1.03	0.848
Central memory T cytotoxic lymphocytes (%)	9.16 (±6.1)	7.80 (±7.6)	1.00	0.349
Effector memory T cytotoxic lymphocytes (%)	39.87 (±15.8)	35.10 (±15.9)	−1.17	0.127
Terminally differentiated T cytotoxic lymphocytes (%)	23.45 (±16.1)	31.60 (±21.4)	1.28	0.238
**Exhausted T cytotoxic lymphocytes (%)**	**6.58 (±4.6)**	**19.95 (±14.5)**	**2.70**	**<0.0001**
Senescent T cytotoxic lymphocytes (%)	5.35 (±5.0)	7.57 (±6.9)	1.28	0.182
**B lymphocytes (%)**	**11.97 (±8.2)**	**18.22 (±7.5)**	**1.61**	**<0.0001**
Natural Killer cells (%)	14.05 (±6.2)	14.17 (±10.1)	−1.06	0.163
IL-12 (pg/mL)	2.00 (±2.1)	1.50 (±1.8)	−1.27	0.484
**TNF (pg/mL)**	**2.88 (±1.8)**	**2.78 (±8.4)**	**−1.18**	**<0.0001**
IL-10 (pg/mL)	2.86 (±1.6)	3.36 (±2.6)	1.19	0.674
IL-6 (pg/mL)	7.93 (±2.8)	34.45 (±60.8)	3.63	0.14
**IL-1β (pg/mL)**	**7.19 (±3.1)**	**2.20 (±2.1)**	**−3.11**	**<0.0001**
IL-8 (pg/mL)	37.50 (±13.5)	40.65 (±47.9)	1.19	0.113

Differences in COVID-19, patients with respect to healthy controls are shown with the Fold change and their statistical significance is shown by the *p*-value assessed by ANCOVA* or non-parametric ANCOVA, analysis including sex as a covariate. Significantly, different variables are highlighted in bold.

Interestingly, we found that the frequency of the exhausted cells, which was measured by the presence of the inhibitory receptor PD-1, is significantly higher in the COVID-19 patient group than in the healthy control group. This significant increase of exhausted cells was found in both T helper (*p* < 0.0001) (Figure 1F) and T cytotoxic cells (*p* < 0.0001) (Figure 1G).

Referring to blood cytokines, in COVID-19 patients there is a significant reduction in TNF (*p* < 0.0001) (Figure 1I) and IL-1β levels (*p* < 0.0001) (Figure 1J).

### 3.2 Immunological changes are more marked among young COVID-19 patients when compared to healthy controls

To gain insight into the effect of age-related host factors in the immune response to COVID-19 and *vice versa*, we analysed the cytokine serum levels and immune cell profiles by age ranges. To do so, four age ranges were stablished: from 30 to 39 years, 40–49 years, 50–59 years and 60–69 years. Additionally, we performed a correlation analysis with age.

**FIGURE 2:**
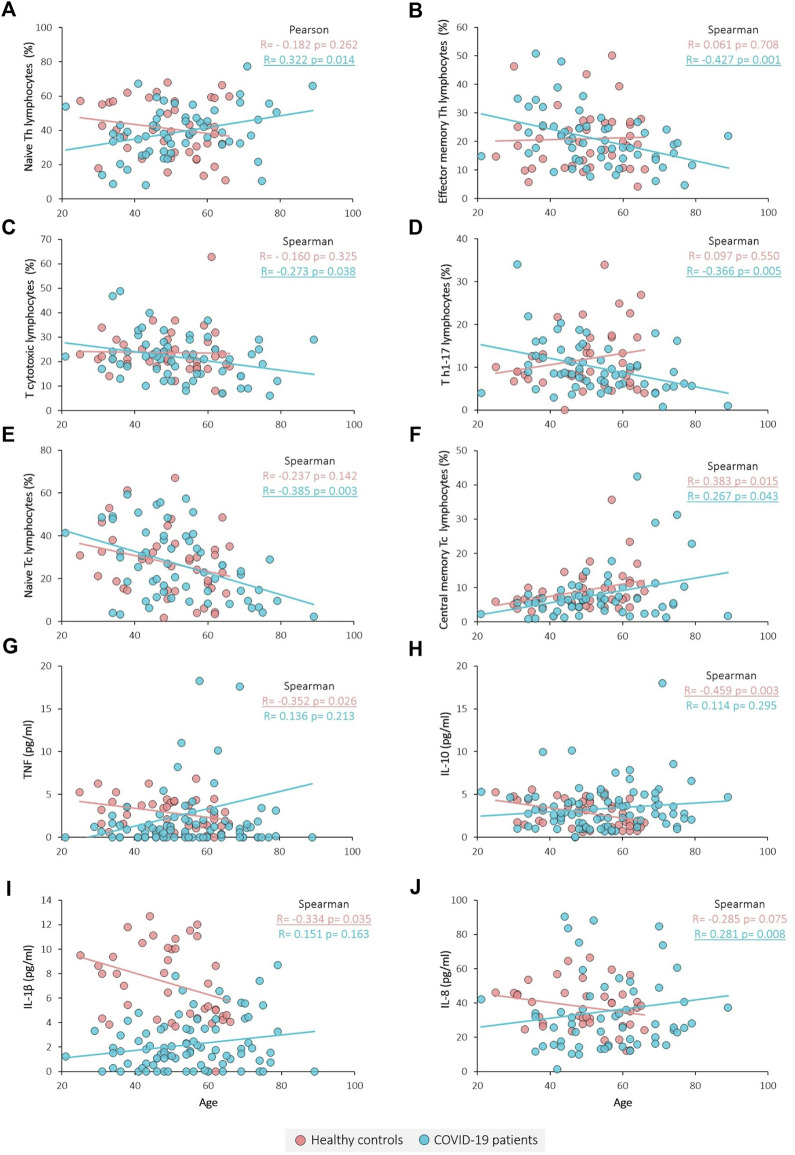
Correlation between immune variables and age. **(A)** Naïve Th lymphocytes percentage, **(B)** Effector memory Th lymphocytes percentage, **(C)** Tc lymphocytes percentage, **(D)** Th1-17 lymphocytes percentage, **(E)** Naïve Tc lymphocytes percentage, **(F)** Central memory Tc lymphocytes percentage, **(G)** TNF concentration, **(H)** IL-10 concentration, **(I)** IL-1β concentration and **(J)** IL-8 concentration. Pearson and Spearman tests were used for normally and non-normally distributed variables, respectively. Significant results are underlined.

Age correlation analysis was performed for each of the groups, showing that some immune variables present different correlation tendencies between groups (Figure 2; Table 5). The proportion of naïve Th lymphocytes is significantly increased with age in patients, whereas controls remain stable (Figure 2A). The same pattern is observed for IL-8 (Figure 2J), while for TNF, IL-10 and IL-1β the decrease in controls was significant and patients remain stable (Figures 2G–I). On the contrary, effector memory T helper lymphocytes and Th17 lymphocytes are found to significantly decrease with age only in patients (Figures 2B, D). In the case of T cytotoxic lymphocytes and particularly for naïve T cytotoxic lymphocytes, there is a negative correlation with age only in COVID-19 patients (Figures 2C, E). Lastly, central memory T cytotoxic lymphocytes are observed to significantly increase with age in both groups (Figure 2F).

**TABLE 5:** Age correlation analysis results. Healthy controls and COVID-19 patients were analysed separately revealing that for most of the variables the age correlation is group specific.

	Healthy controls		COVID-19 patients	
	R	*p*-value	R	*p*-value
Total leukocytes (%)	−0,102	0,532	−0,19	0,153
T lymphocytes (%)	−0,289	0,071	−0,065	0,63
T helper lymphocytes (%)	−0,095	0,559	0,247	0,061
Naive T helper lymphocytes (%)*	−0,182	0,262	**0,322**	**0,014**
Central memory T helper lymphocytes (%)	0,204	0,208	0,042	0,752
**Effector memory T helper lymphocytes (%)**	0,061	0,708	**−0,427**	**0,001**
Terminally differentiated T helper lymphocytes (%)	−0,166	0,306	−0,002	0,988
T helper type 1 lymphocytes (Th1) (%)	0,018	0,914	0,033	0,807
T helper type 2 lymphocytes (Th2) (%)	0,301	0,059	−0,125	0,349
T helper type 17 lymphocytes (Th17) (%)	0,177	0,282	−0,111	0,407
**T helper type 1–17 lymphocytes (Th1-17) (%)**	0,097	0,55	**−0,366**	**0,005**
Regulatory T lymphocytes (%)	0,117	0,472	0,079	0,555
Exhausted T helper lymphocytes (%)	−0,066	0,684	0,072	0,59
Senescent T helper lymphocytes (%)	0,046	0,808	0,088	0,512
**T cytotoxic lymphocytes (%)**	−0,16	0,325	**−0,273**	**0,038**
**Naive T cytotoxic lymphocytes (%)**	−0,237	0,142	**−0,385**	**0,003**
**Central memory T cytotoxic lymphocytes (%)**	**0,383**	**0,015**	**0,267**	**0,043**
Effector memory T cytotoxic lymphocytes (%)	0,148	0,361	0,064	0,632
Terminally differentiated T cytotoxic lymphocytes (%)	−0,095	0,561	−0,014	0,917
Exhausted T cytotoxic lymphocytes (%)	0,179	0,268	0,235	0,076
Senescent T cytotoxic lymphocytes (%)	−0,115	0,545	0,009	0,947
B lymphocytes (%)	0,178	0,272	−0,007	0,956
Natural Killer cells (%)	0,25	0,12	0,079	0,555
**IL-12 (pg/mL)**	**−0,367**	**0,2**	0,102	0,347
**TNF (pg/mL)**	**−0,352**	**0,026**	0,136	0,213
IL-10 (pg/mL)	−0,459	0,003	0,114	0,295
IL-6 (pg/mL)	−0,288	0,071	0,148	0,177
**IL-1β (pg/mL)**	**−0,334**	**0,035**	0,151	0,163
**IL-8 (pg/mL)**	−0,285	0,075	**0,281**	**0,008**

R correlation coefficient and *p*-values assessed by Pearson* or Spearman are shown. Significantly different variables are highlighted in bold.

The analysis per ranges showed 10 significantly different variables between COVID-19 patients and healthy controls for the youngest age range analyzed, which included those aged between 30–39 years. Less significant changes were identified for the rest of the age ranges (Table 6).

**TABLE 6:** Statistical analysis of immune cell populations and cytokine concentration results by age ranges. Mean results are shown for each of the variables and groups.

	30–39 years				40–49 years				50–59 years				60–69 years			
	HC	Patients	FC	*p*-value	HC	Patients	FC	*p*-value	HC	Patients	FC	*p*-value	HC	Patients	FC	*p*-value
Total leukocytes (%)	39,16	18,73	−2,09	0,44	30,13	33,06	1,10	0,701	**41,40**	**23,43**	**−1,77**	**<0,0001**	**36,24**	**27,27**	**−1,33**	**0,006**
T lymphocytes (%)	75,80	67,42	−1,12	0,177	76,30	69,81	−1,09	0,293	74,28	67,45	−1,10	0,126	70,98	67,69	−1,05	0,614
T helper lymphocytes (%)	**49,34**	**39,55**	**−1,25**	**0,041**	43,85	44,68	1,02	0,618	49,89	46,09	−1,08	0,389	49,23	46,50	−1,06	0,773
Naive T helper lymphocytes (%)*	**44,71**	**27,72**	**−1,61**	**0,027**	39,20	39,77	1,01	0,220	33,63	41,46	1,23	0,082	41,98	43,39	1,03	0,967
Central memory T helper lymphocytes (%)	30,59	36,72	1,20	0,477	36,24	36,13	−1,00	0,394	35,31	39,30	1,11	0,114	37,23	35,92	−1,04	0,990
Effector memory T helper lymphocytes (%)	**20,01**	**29,47**	**1,47**	**0,034**	23,02	21,58	−1,07	0,343	**26,54**	**17,53**	**−1,51**	**0,034**	17,89	16,68	−1,07	0,678
Terminally differentiated T lympho. (%)	4,70	6,04	1,29	0,412	4,81	2,96	−1,62	0,740	4,47	1,72	−2,60	0,167	2,97	4,16	1,40	0,329
T helper type 1 lymphocytes (Th1) (%)	19,89	16,84	−1,18	0,783	18,50	13,70	−1,35	0,746	21,23	15,70	−1,35	0,111	17,25	16,46	−1,05	0,813
T helper type 2 lymphocytes (Th2) (%)	**19,72**	**30,21**	**1,53**	**0,027**	23,36	26,56	1,14	0,350	22,46	22,93	1,02	0,460	22,55	24,54	1,09	0,778
T helper type 17 lymphocytes (Th17) (%)	6,80	10,74	1,58	0,068	10,07	11,16	1,11	0,087	7,85	9,00	1,15	0,439	8,08	11,27	1,39	0,098
T helper type 1–17 lympho. (Th1-17) (%)	9,35	15,29	1,63	0,077	11,68	10,73	−1,09	0,311	**15,20**	**8,82**	**−1,72**	**0,022**	11,82	8,50	−1,39	0,395
Regulatory T lymphocytes (%)	3,42	2,68	−1,28	0,107	3,79	3,31	−1,14	0,201	**4,12**	**3,54**	**−1,16**	**0,466**	**3,90**	**3,67**	**−1,06**	**0,254**
Exhausted T helper lymphocytes (%)	**2,51**	**10,28**	**4,10**	**<0,0001**	4,50	6,82	1,51	0,068	**1,81**	**10,62**	**5,86**	**<0,0001**	**2,62**	**10,14**	**3,88**	**0,001**
Senescent T helper lymphocytes (%)	4,03	2,36	−1,71	0,582	1,25	1,81	1,45	0,709	3,71	1,21	−3,06	0,753	1,52	2,26	1,49	0,526
T cytotoxic lymphocytes (%)	23,17	25,72	1,11	0,931	28,38	22,72	−1,25	0,752	22,19	19,23	−1,15	0,149	23,66	19,42	−1,22	0,536
Naive T cytotoxic lymphocytes (%)	32,74	33,17	1,01	0,839	23,99	30,25	1,26	0,266	30,39	29,64	−1,03	0,390	22,23	17,98	−1,24	0,384
Central memory Tc lymphocytes (%)	6,08	4,81	−1,27	0,354	8,65	5,84	−1,48	0,342	9,66	9,14	−1,06	0,286	11,19	11,94	1,07	0,097
Effector memory Tc lymphocytes (%)	34,64	30,43	−1,14	0,806	51,10	33,70	−1,52	0,078	37,75	39,43	1,04	0,865	41,50	27,76	−1,49	0,067
Terminally differentiated Tc lympho. (%)	26,28	36,95	1,41	0,49	16,43	30,17	1,84	0,307	20,43	21,80	1,07	0,934	25,29	42,34	1,67	0,173
Exhausted T cytotoxic lymphocytes (%)	**5,14**	**15,24**	**2,96**	**0,001**	6,66	14,86	2,23	0,205	**6,50**	**21,63**	**3,33**	**0,039**	7,70	18,22	2,37	0,064
Senescent T cytotoxic lymphocytes (%)	4,46	7,99	1,79	0,638	7,26	7,94	1,09	0,671	4,84	4,68	−1,04	0,674	5,82	8,90	1,53	0,146
B lymphocytes (%)	9,86	18,91	1,92	0,017	16,33	16,75	1,03	0,200	**12,24**	**19,56**	**1,60**	**0,039**	**10,81**	**17,62**	**1,63**	**0,040**
Natural Killer cells (%)	14,10	13,79	−1,02	0,501	11,60	13,49	1,16	0,967	13,40	13,51	1,01	0,923	17,87	16,61	−1,08	0,382
Interleukine-12 (pg/mL)	2,07	0,90	−2,30	0,237	**2,85**	**0,91**	**−3,12**	**0,037**	2,27	1,44	−1,58	0,581	**0,88**	**2,11**	**2,40**	**0,048**
Tumor Necrosis Factor (TNF) (pg/mL)	**3,11**	**1,26**	**−2,47**	**0,01**	**3,27**	**0,79**	**−4,14**	**<0,0001**	2,76	2,90	1,05	0,243	1,61	7,26	4,51	0,892
Interleukine-10 (pg/mL)	3,25	3,52	1,08	0,863	3,14	2,74	−1,15	0,342	3,44	2,65	−1,30	0,242	**1,75**	**3,78**	**2,16**	**0,001**
Interleukine-6 (pg/mL)	7,66	33,29	4,35	0,369	17,23	17,81	1,03	0,177	7,37	21,19	2,87	0,935	**6,69**	**24,65**	**3,69**	**0,012**
Interleukine-1β (pg/mL)	**7,32**	**1,51**	**−4,86**	**<0,0001**	**8,68**	**1,59**	**−5,45**	**<0,0001**	**8,02**	**2,57**	**−3,12**	**<0,0001**	**4,81**	**2,16**	**−2,23**	**0,022**
Interleukine-8 (pg/mL)	**37,68**	**24,10**	**−1,56**	**0,02**	48,80	29,27	−1,67	0,116	38,93	34,33	−1,13	0,396	30,26	46,14	1,52	0,817

Differences in COVID-19, patients with respect to healthy controls are shown with the fold change and their statistical significance is shown by the *p*-value assessed by non-parametric ANCOVA, analysis including sex as a covariate. Significantly different variables are highlighted in color.

**FIGURE 3:**
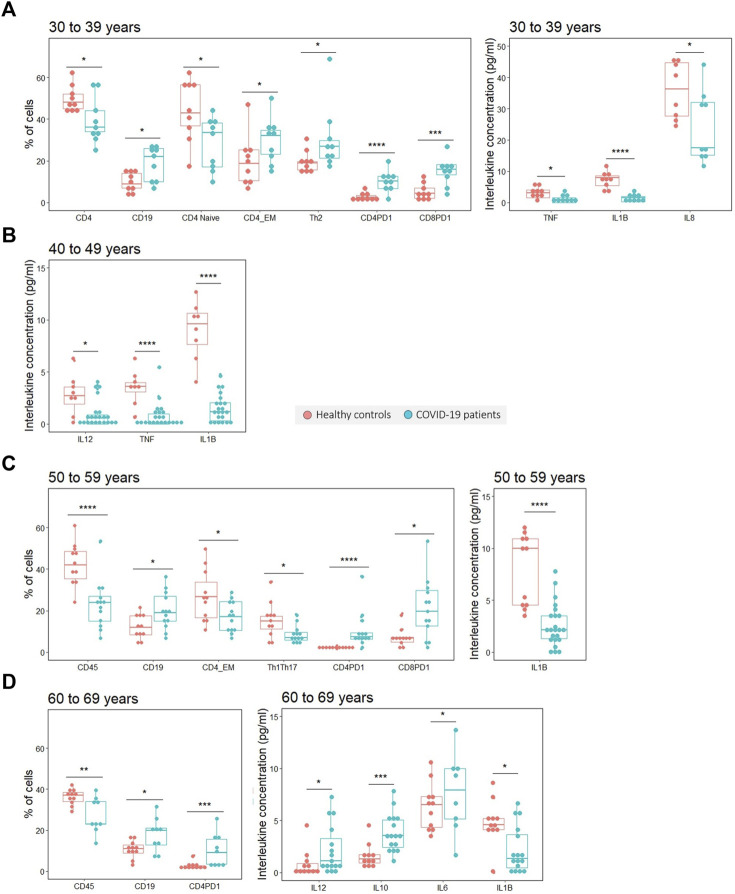
Variables found to be significantly different in COVID-19 patients with respect to healthy controls analyzed by age ranges. **(A)** Significantly different variables in the age range from 30 to 39 years old. **(B)** Significantly different variables in the age range from 40 to 49 years old. **(C)** Significantly different variables in the age range from 50 to 59 years old. **(D)** Significantly different variables in the age range from 60 to 69 years old. The statistical significance is depicted with asterisk code meaning: *p < 0.05; **p < 0.01; ***p < 0.001; ****p < 0.0001.

Among those variables that are significantly different only between individuals from 30 to 39 years we found T helper lymphocytes (*p* = 0.041), naïve T helper lymphocytes (*p* = 0.027) and IL-8 (*p* = 0.02), which are decreased in young COVID-19 patients and Th2 lymphocytes which are significantly increased (*p* = 0.027) (Figure 3A). In this line, TNF is only significantly decreased in those patients under 49 years (age ranges 30–39 and 40–49) (Table 6) (Figures 3A, B).

On the contrary, and although less in number, there are some variables which are more significantly affected in older individuals. The increase in IL-10 (*p* = 0.001) and IL-6 (*p* = 0.012) is exclusively observed in the oldest age range, which included individuals between 60 and 69 years (Figure 3D), and a significant decrease in leukocytes is found only in patients older than 50 years (age ranges 50–59 and 60–69) (Table 6) (Figures 3C, D).

Interestingly, some other immune variables show differences regardless of age with significant results in all or at least three different age ranges. IL-1β for example is found to be significantly decreased in the four age ranges. There is also an increase in B lymphocyte proportion in all age ranges except for those between 40 and 49 years in which there is no difference. Lastly, significant differences in some other variables, such as the proportion of effector memory T helper lymphocytes and the levels of IL-12 are found in particular or separated age ranges (Table 6)(Figure 3).

To finish, it is worth noting that in line with what we found in the whole cohort analysis, COVID-19 patients show a higher proportion of exhausted T helper and T cytotoxic lymphocytes in most age ranges. Exhausted T helper lymphocytes are significantly increased in patients aged 30 to 39 (*p* < 0.0001), 50 to 59 (*p* < 0.0001) and 60–69 years (*p* = 0.001), and exhausted T cytotoxic lymphocytes in 30–39 (*p* = 0.001) and 50–59 years (*p* = 0.039) (Table 6) (Figure 3).

## 4 Discussion

In this study, we described the immune changes found in COVID-19 patients when compared to healthy controls that were not infected. We analyzed blood samples of patients when they were admitted to the hospital. We are aware of the different times of COVID-19 diagnosis (with some patients being diagnosed even before any symptoms arise, while others are only confirmed when they develop symptoms). Aiming to establish a similar point for all patients, we decided to analyze immune cells and cytokines when patients met the criteria for being admitted to the hospital.

In line with what has been previously described by others (Del Valle et al., 2020; Hadjadj et al., 2020; Huang and Pranata, 2020; Laing et al., 2020; Lucas et al., 2020; Ronit et al., 2020; Caricchio et al., 2021; Masso-Silva et al., 2022), COVID-19 patients in our cohort had lymphopenia. A significant reduction in T lymphocytes is also observed and a decrease of Th and Tc lymphocytes was found in the whole cohort only in absolute cell numbers. In addition, both exhausted Th and Tc lymphocytes are increased in COVID-19 patients. Moreover, although there is no difference in the proportion of NK cells, a reduced absolute number of NK cells in patients was found, suggesting that they may have a partially impaired innate immune response.

Although lymphopenia in T cell has been extensively described in COVID-19 patients, only few works studied B cell proportions. Some studies found an increase in this population associated with COVID-19 disease severity (Wang et al., 2020; Bobcakova et al., 2021), while on the contrary, other works described a decrease in B cell with severity (Hadjadj et al., 2020; Çölkesen et al., 2022). Regarding B lymphocyte proportions, Qin et al. described that B cell were within the lower level of the normal range, but there was no significant difference between COVID-19 patients and healthy controls (Qin et al., 2020). On the contrary, our results show a significant increase of B lymphocytes in COVID-19 patients compared to healthy controls. Therefore, the results for B cell in COVID-19 literature are heterogeneous and need further investigation.

Related to the hyperinflammatory phase described for COVID-19, a characteristic cytokine storm has been reported and associated with disease severity and mortality. Mt. Sinai Hospital performed the first extensive study documenting cytokine levels of 1,484 patients and found elevated serum IL-6, IL-8 and TNF concentrations compared to those with negative PCRs (Del Valle et al., 2020). Moreover, they reported that high serum levels of these three cytokines at the time of admission were able to predict poor outcomes (Del Valle et al., 2020). In addition, other studies reported that IL-1β was also increased in COVID-19 patients and in particular in severe cases (Lucas et al., 2020; Masso-Silva et al., 2022). In our cohort, for IL-6 and IL-8 we did not find a significant increase compared to controls. Moreover, in contrast to what has been described, levels of TNF and IL-1β are found to be significantly reduced in our whole cohort of COVID-19 patients.

These results could indicate that at the moment of drawing the samples for our study, patients did not have hyperinflammation, but they were at a milder phase of the disease with little evidence of hyperinflammation. This reduced pro-inflammatory response, and in particular the reduction in IL-1β, could also be linked to a reduced activation of the innate and adaptive cells that are crucial during the antiviral defense. Although hyperinflammation is one of the best-known hallmarks of severe COVID-19, in line with our results, some other studies have also found that this hyperinflammation may not be present in all patients and phases of the disease. In fact, studies in which different cytokine blocking treatments have been tried for COVID-19 patients, found that patients with mild-COVID-19 do not benefit from the treatment as those with severe COVID-19 did (Cavalli et al., 2020; Huet et al., 2020; Mariette et al., 2021).

On the other hand, the immune system is known to be changed with age as well as with prolonged chronic infections or immune diseases that may accelerate the natural aging of the immune system (Nikolich-Žugich, 2018). In addition, the aging of the immune system has been associated with a decreased capability of response and with increased vulnerability to infections and worse outcomes (Kauffman, 2004). Thus, immunosenescence may be one of the reasons why older people get more severe COVID-19. It should be mentioned that the correlation analysis of our cohort did not show significant results in some of the characteristic signs of immunosenescence with aging (Aiello et al., 2019). This was not the focus of our work, but we expected to see these alterations with age, at least in the control group. However, healthy donors were <70 years and, therefore, the lack of older donors could have limited the correlation analysis. Additionally, we also hypothesize that an accelerated immunosenescence could be the reason why, although most young people are asymptomatic (Red Nacional de Vigilancia Epidemiológica, 2022), some of them develop symptomatic COVID-19 and are hospitalized. In order to assess these questions, and to get more specific results, we performed subgroup analysis by separating all the individuals in 10-year age ranges going from 30 to 69 years.

In a previous meta-analysis study, Huang et al. also performed a subgroup analysis using 55 years old as cutoff point, and reported that there was an effect of age in lymphocytes (Huang and Pranata, 2020). Moreover, they suggested that the aging of the immune system could contribute to a relatively “non-reactive” immune state in older patients causing less pronounced changes (Huang and Pranata, 2020). Nevertheless, their main finding was that the association between lymphopenia and COVID-19 was stronger in younger patients whereas, in our cohort, we see a significant lymphopenia only in patients older than 50. These discrepancies could be due to the age heterogeneity between studies as well as to different definitions of lymphopenia (Huang and Pranata, 2020).

Our most remarkable finding regarding the analysis by age ranges is that the differences between COVID-19 patients and healthy controls were bigger in the age range between 30–39 years than for the rest of the age ranges. To our mind, two different interpretations of this finding can be done. The first hypothesis is that those young individuals with a worse COVID-19 outcome, may have a different immune condition prior to the infection. The second hypothesis is in agreement with Huang et al. (Huang and Pranata, 2020) suggesting that a young immune system responds more strongly to the SARS-CoV-2 infection compared to the response mounted by older patients, resulting in greater differences between young patients and controls.

A reduction in the proportion of T helper lymphocytes has been suggested as a feature of COVID-19 disease (Zheng et al., 2020b; Giamarellos-Bourboulis et al., 2020; Huang et al., 2020; Lucas et al., 2020; Gustine and Jones, 2021), which could indicate the exhaustion of the immune system after the infection. This suggests that, after an initial response, not only the antiviral T cell response, but also the Th dependent B cell response and thus the antibody response that is involved in virus neutralization, opsonization and antibody dependent cytotoxicity are progressively reduced. When analyzing the whole cohort, we did not find a global reduction in the proportion of T helper cells, but this difference is significant in the analysis performed between 30 and 39 years. This finding together with a sharp increase in the proportion of Th and Tc cells expressing the PD-1 marker indicate that T cell response is exhausted in young COVID-19 patients. On the other hand, the decrease in naïve T helper lymphocytes, that has been associated with immunosenescence (Kim et al., 2008; Pawelec, 2012), could reinforce the idea of a premature aging of the immune system in young COVID-19 patients that could be the reason for, or the consequence of developing a moderate to severe course of the disease in young people.

Previous works have linked age with B cell response and antibody titers in COVID-19. Rydyznski Moderbacher and colleages described that the coordination of SARS-CoV-2 antigen-specific responses was disrupted in individuals older than 65 years (Rydyznski Moderbacher et al., 2020). On the contrary, our correlation analysis show that B cell do not decrease with age. Moreover, when the proportion of B lymphocytes was compared between COVID-19 patients and controls, an increase in these cells was found in 3 of our age ranges. This suggest an elevated antibody-mediated immune response maintained with age.

Regarding inflammatory cytokines, it is worth noting that the concentration of TNF, IL-1β and IL-8 is significantly decreased among the youngest patients. In contrast, only patients between 50 and 60 years showed a significant increase in IL-6, IL-12 and IL-10 when compared to controls. Interestingly, an increase in TNF and IL-6 have also been described as features present in inflammaging (Michaud et al., 2013; Alberro et al., 2021).

Therefore, our findings in the context of other previous studies, suggest that aging affects the behavior of the immune system in COVID-19 patients. They may indicate that young individuals are able to mount an initial response to SARS-CoV-2, but some of them present an accelerated exhaustion of the cell response and an insufficient inflammatory response, resulting in a moderate to severe COVID-19 (Cunha et al., 2020; Domingues et al., 2020). On the other hand, our results could suggest that in older patients there is a smaller immune cell response to the virus, reflected in fewer differences in immune populations between patients and controls. Nevertheless, old patients show more evidence of an inflammatory phenotype suggesting that the underlying inflammation associated with their age is exacerbated by the SARS-CoV-2 infection.

## Data Availability

The original contributions presented in the study are included in the article/Supplementary Material, further inquiries can be directed to the corresponding author.
