# Supplementary material for: Immune cell population and cytokine profiling suggest age dependent differences in the response to SARS-CoV-2 infection
Source: Front Aging. 2023 Feb 13;4:1108149. doi: 10.3389/fragi.2023.1108149 (PMC9968858; doi:10.3389/fragi.2023.1108149)
Supplement: Supplementary file 1 [file DataSheet1.PDF]

Supplementary Table 1. List of immune cell populations analysed by flow cytometry and their corresponding markers and antibodies. Note that they have been analyzed in five different panels.

**Panel 1**<br>**Absolute leukocytes**	**Panel 2**<br>**Effector and memory T lymphocytes**	**Panel 3**<br>**T helper lymphocyte subtypes**	**Panel 4**<br>**Regulatory T lymphocytes**	**Panel 5**<br>**Immunosenescence**
Anti-CD45 – PerCP-Cy5.5<br>Clone 2D1 (HLe-1)24<br>Becton Dickinson	Anti-CD45 – V500<br>Clone HI30<br>Becton Dickinson	Anti-CD45 – V500<br>Clone HI30<br>Becton Dickinson	Anti-CD45 – V500<br>Clone HI30<br>Becton Dickinson	Anti-CD45 – V500<br>Clone HI30<br>Becton Dickinson
Anti-CD3 – FITC<br>Clone SK716-19<br>Becton Dickinson	Anti-CD3 – V450<br>Clone UCTH1<br>Becton Dickinson	Anti-CD3 – V450<br>Clone UCTH1<br>Becton Dickinson	Anti-CD3 – V450<br>Clone UCTH1<br>Becton Dickinson	Anti-CD3 – V450<br>Clone UCTH1<br>Becton Dickinson
Anti-CD4 - Pe-Cy7<br>Clone SK325-27<br>Becton Dickinson	Anti-CD4 - PerCP-Cy5<br>Clone SK3<br>Becton Dickinson	Anti-CD4 - PerCP-Cy5<br>Clone SK3<br>Becton Dickinson	Anti-CD4 - PerCP-Cy5<br>Clone SK3<br>Becton Dickinson	Anti-CD4 - PerCP-Cy5<br>Clone SK3<br>Becton Dickinson
Anti-CD8 – APC- Cy7<br>Clone SK125-26<br>Becton Dickinson	Anti-CD8 – APC-H7<br>Clone SK1<br>Becton Dickinson	Anti-CD8 – APC-H7<br>Clone SK1<br>Becton Dickinson	Anti-CD8 – APC-H7<br>Clone SK1<br>Becton Dickinson	Anti-CD8 – APC-H7<br>Clone SK1<br>Becton Dickinson
Anti-CD19 – APC<br>Clone SJ25C128<br>Becton Dickinson	Anti-CD45RA – PE-Cy7<br>Clone L48<br>Becton Dickinson	Anti-CD196 (CCR6) - PE<br>Clone 11A9<br>Becton Dickinson	Anti-CD25 – PE<br>Clone 2A3<br>Becton Dickinson	Anti-CD56 – PE-Cy7<br>Clone NCAM16.2<br>Becton Dickinson
Anti-CD56/16 – PE<br>Clone NCAM16.223/<br>B73.120-22<br>Becton Dickinson	Anti-CD197 (CCR7) –<br>PE<br>Clone 3D12<br>Becton Dickinson	Anti-CD183 (CXCR3) -<br>APC<br>Clone G025H7<br>BioLegend	Anti-CD127 - APC<br>Clone A019D5<br>BioLegend	Anti-CD28 –APC<br>Clone CD28.2<br>Becton Dickinson
				Anti-CD279 -FITC<br>Clone HNK1<br>Becton Dickinson
				Anti-CD57 - PE<br>Clone MIH4<br>Becton Dickinson

Supplementary Table 2. Statistical analysis of the immune cell populations analyzed using BD Multitest™ 6-color TBNK Reagent kit with BD Trucount tubes.

	**Healthy Controls**	**COVID-19 patients**	**Fold Change**	**p-value**
**T lymphocytes (cells/µl)**	**1476.0±537.2**	**883.4±548.1**	**-1.67**	**<0,0001**
**T helper lymphocytes (cells/µl)**	**994.6±464.91**	**586.7±425.4**	**-1.70**	**<0,0001**
**T cytotoxic lymphocytes (cells/µl)**	**449.0±170.5**	**278.9±167.9**	**-1.60**	**<0,0001**
B lymphocytes (cells/µl)	220.4±125.1	238.8±205.3	1.08	0.796
**Natural Killer cells (cells/µl)**	**283.3±156.3**	**153.8±118.9**	**-1,84**	**<0,0001**

Mean and standard deviation from the absolute cell count are shown for each of the variables and groups.
