# Supplementary material for: Immune cell population and cytokine profiling suggest age dependent differences in the response to SARS-CoV-2 infection
Source: Front Aging. 2023 Feb 13;4:1108149. doi: 10.3389/fragi.2023.1108149 (PMC9968858; doi:10.3389/fragi.2023.1108149)

Supplementary Figure 1. Representation of flow cytometry gating strategy for immunological cell populations. (A) Gating strategy for the variables analyzed with the BD Multitest™ 6-color TBNK Reagent kit. **(B)** This figure collects and outlines the gating strategy followed in the four multicolored FC panels.

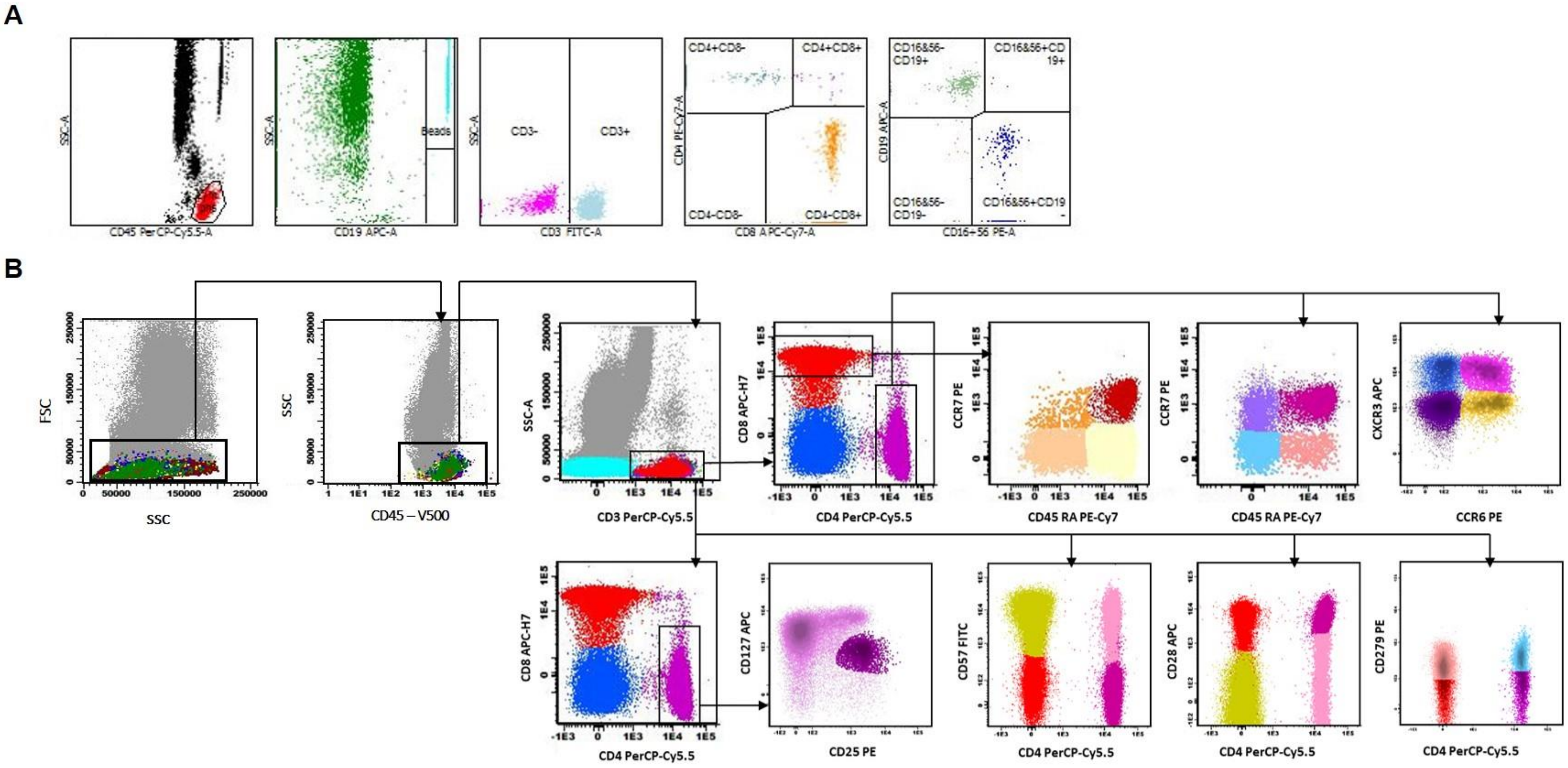

Supplement: Supplementary file 2 [file Image1.pdf]
